# Molecular Mechanisms of Photobiomodulation in Retinal Diseases: Cytochrome c Oxidase, Mitochondrial Bioenergetics and Cytoprotective Signalling

**DOI:** 10.3390/ijms27135683

**Published:** 2026-06-24

**Authors:** Rubens Camargo Siqueira

**Affiliations:** 1 Rubens Siqueira Research Center, São José do Rio Preto 15010-100, SP, Brazil; siqueiraretina@gmail.com; 2 Postgraduate Program, Faculty of Medicine of São José do Rio Preto—FAMERP, São José do Rio Preto 15010-100, SP, Brazil

**Keywords:** photobiomodulation, cytochrome c oxidase, mitochondria, circadian rhythm, Nrf2, NF-κB, PGC-1α, BMAL1, mitochondrial biogenesis, mitophagy, age-related macular degeneration, retinitis pigmentosa, central serous chorioretinopathy, myopia, near-infrared light

## Abstract

Photobiomodulation (PBM) is a non-invasive therapeutic strategy that uses red and near-infrared (NIR) light in the 590–950 nm range to modulate the cellular and molecular pathways involved in retinal homeostasis. At the molecular level, PBM acts primarily through photon absorption by cytochrome c oxidase (CcO, complex IV of the mitochondrial electron transport chain), whose four metal centres—two copper (CuA and CuB) and two heme groups (heme a and heme a_3_)—absorb light across approximately 600–1000 nm. Photon capture promotes photodissociation of inhibitory nitric oxide (NO) from the binuclear CuB–heme a_3_ centre, accelerates electron transfer, restores the proton-motive force and increases ATP synthesis. These primary events trigger a coordinated molecular programme that includes (i) transient mitochondrial reactive oxygen species (ROS) bursts that activate the Nrf2/Keap1/ARE axis and upregulate phase II antioxidant enzymes (HO-1, NQO1, GCLC, SOD2, catalase, GPx); (ii) calcium- and cAMP-dependent secondary signalling that converges on PI3K/Akt, MAPK/ERK, AMPK and mTOR pathways; (iii) suppression of NF-κB-driven cytokine production (TNF-α, IL-1β, IL-6) and of NLRP3 inflammasome activation; (iv) downregulation of the HIF-1α/VEGF axis, particularly at 590 nm; (v) anti-apoptotic remodelling of the Bcl-2/Bax ratio with reduced cytochrome c release and caspase-3/9 activation; and (vi) PGC-1α/TFAM/NRF1-driven mitochondrial biogenesis, alongside restoration of fission/fusion homeostasis (Drp1, Mfn1/2, Opa1) and PINK1/Parkin-mediated mitophagy. Wavelength specificity has a defined molecular basis: 590 nm modulates VEGF signalling and RPE pump activity, 660 nm interacts with the CuB centre and enhances O_2_ binding at CcO, and 850 nm is absorbed by CuA and supports electron entry into complex IV. A second molecular axis is the bidirectional crosstalk between PBM and the circadian system: mitochondrial respiration, ATP turnover and CcO activity oscillate over the 24 h cycle under the control of the BMAL1/CLOCK and PER/CRY core machinery, the NAD^+^/SIRT1–SIRT3 axis and REV-ERBα. Preliminary preclinical and human observations suggest that NIR-induced bioenergetic and functional gains may be coupled to this rhythm, with greater benefit reported when light is delivered in the morning window (≈08:00–11:00); this time dependence should be regarded as an emerging hypothesis rather than an established clinical principle. The clinical evidence is unevenly developed across indications. It is most robust for non-exudative age-related macular degeneration, where multiwavelength PBM (590/660/850 nm; Valeda Light Delivery System) has shown disease-modifying potential in randomized controlled trials (LIGHTSITE I–III and the LIGHTSITE IIIB extension), with sustained BCVA gains and reduced incidence of geographic atrophy over 24 months and beyond. Evidence for retinitis pigmentosa, central serous chorioretinopathy and, with red-light monotherapy, childhood myopia is at present limited to small or short-term studies and remains preliminary. This narrative review synthesizes the molecular machinery engaged by PBM, integrates clinical findings across retinal diseases and discusses how chronotherapeutic delivery of light, aligned with the molecular clock, may further optimize therapeutic efficacy.

## 1. Introduction

The retina is one of the most metabolically demanding tissues in the human body. Photoreceptors, the retinal pigment epithelium (RPE) and the inner retinal neurons exhibit very high rates of oxygen consumption and ATP turnover, which makes them particularly vulnerable to mitochondrial dysfunction and oxidative damage. Mitochondrial decline is now recognized as a central pathogenic mechanism in age-related macular degeneration (AMD), several inherited retinal dystrophies, central serous chorioretinopathy (CSCR) and myopia-associated retinal stress, where it contributes to oxidative imbalance, chronic inflammation, RPE dysfunction and photoreceptor death [1,2].

Photobiomodulation (PBM), also known as low-level light therapy, is a non-thermal, non-invasive intervention that delivers red and near-infrared (NIR) light—typically between 500 and 1000 nm—to biological tissues to stimulate cellular repair, restore mitochondrial bioenergetics and modulate inflammation [3,4]. Light-emitting diodes (LEDs) are preferred over coherent lasers for ocular applications because they irradiate larger retinal areas at low fluence, with no thermal damage and no risk of ocular accidents [4].

Over the past decade, PBM has progressed from preclinical models of retinal injury to randomized clinical trials in dry AMD. The LIGHTSITE programme has produced the largest body of controlled evidence to date, culminating in the November 2024 U.S. FDA authorization of the Valeda^®^ Light Delivery System (590/660/850 nm) as the first device-based treatment for dry AMD. In parallel, growing evidence supports PBM in retinitis pigmentosa (RP), CSCR and pathological myopia [5,6,7,8,9,10].

Two molecularly grounded themes are emerging as crucial for moving PBM toward a more mechanism-based, and potentially individualized, retinal therapy. The first is the molecular specificity of the photon–chromophore interaction at cytochrome c oxidase (CcO) and the downstream signalling networks it ignites: mitochondrial bioenergetics, redox-sensitive transcription, calcium- and kinase-mediated secondary messaging, anti-apoptotic remodelling, mitochondrial biogenesis and mitophagy, and modulation of VEGF and inflammatory cascades. The second is chronobiology: mitochondrial function exhibits robust circadian oscillations under the control of a well-defined molecular clock, and preliminary preclinical and human observations suggest that PBM efficacy may depend on the time of day at which light is delivered [11,12,13,14]. Together, these dimensions point toward a chronotherapeutic, mechanism-based approach to PBM. The present review summarizes current molecular evidence—with particular emphasis on the signalling pathways and transcription factors engaged downstream of CcO photoactivation—integrates clinical findings across the main retinal indications, and discusses how circadian considerations may further refine treatment protocols. Throughout, we have sought to distinguish findings that rest on direct experimental evidence from mechanisms that remain inferred or proposed and to reflect the differing levels of clinical evidence across indications.

**Approach to the literature.** This is a narrative, rather than systematic, review. To assemble the evidence base, we searched PubMed/MEDLINE, Scopus and Google Scholar for articles published up to 2025, using combinations of the terms photobiomodulation, low-level light therapy, near-infrared light, cytochrome c oxidase, mitochondria, circadian rhythm, retina, retinal pigment epithelium, age-related macular degeneration, retinitis pigmentosa, central serous chorioretinopathy and myopia. We prioritized peer-reviewed primary studies, with particular weight given to randomized controlled trials and to mechanistic studies with direct experimental readouts, and supplemented these with key reviews and conference reports where primary data were not yet available. Preference was given to the most robust and most recent evidence for each indication; case reports and small single-arm series were included where higher-level evidence was lacking and are identified as such in the text. Because the selection and emphasis reflect the authors’ judgement rather than a prespecified protocol, this review should be read as an interpretive synthesis rather than an exhaustive or unbiased enumeration of the literature.

## 2. Molecular Mechanisms of Photobiomodulation

### 2.1. Cytochrome c Oxidase: Photophysics and Photochemistry of Primary Photoacceptor

The dominant molecular target of red/NIR light in the retina is cytochrome c oxidase (CcO), the terminal complex (complex IV) of the mitochondrial electron transport chain (ETC). CcO is a multimeric heme–copper oxidase that catalyzes the four-electron reduction of molecular oxygen to water, coupled to proton translocation across the inner mitochondrial membrane. The enzyme contains four redox-active metal centres distributed across two functional domains: a binuclear copper centre (CuA) located near the cytochrome c docking site, which serves as the electron entry point; a low-spin heme group (heme a), which transfers electrons from CuA to the catalytic site; and a binuclear active centre composed of a high-spin heme (heme a_3_) and a single copper ion (CuB), where O_2_ is bound and reduced [4,15]. These metal centres collectively absorb light across the ~600–1000 nm range, but with discrete absorption maxima that define the wavelength dependence of PBM. Red light around 660 nm is preferentially absorbed by the reduced CuB centre and enhances oxygen affinity at the active site, whereas NIR light around 810–850 nm is absorbed predominantly by the oxidized CuA centre, promoting intramolecular electron transfer from cytochrome c into the enzyme [4,15]. Throughout this section, we distinguish, as far as the evidence allows, mechanisms supported by direct experimental data from those that are inferred or remain proposed. The identity of CcO as the principal photoacceptor and the resulting rise in ATP are well supported; several of the specific downstream wavelength assignments and the precise photochemistry at the metal centres are more provisional and, in places, still debated.

**Figure 1:**
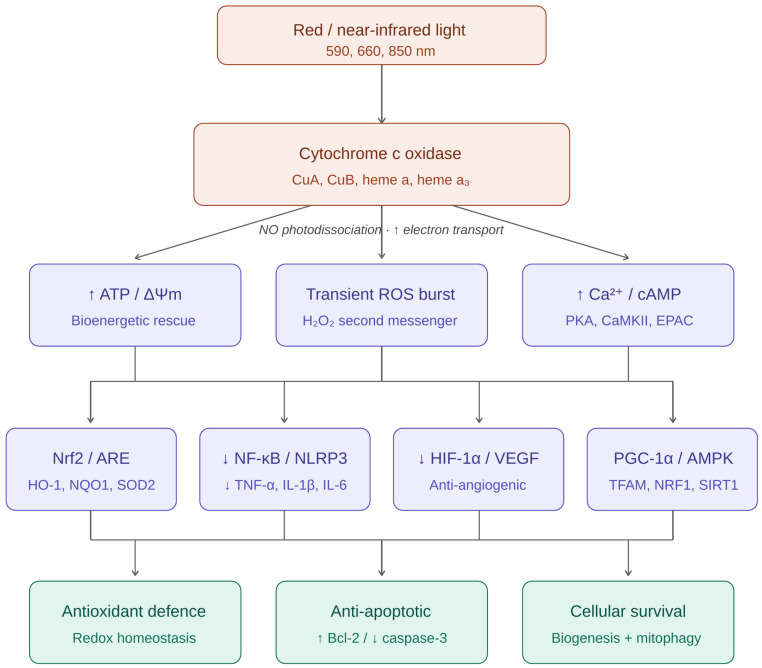
The molecular pathway of photobiomodulation in the retina, from photon capture to cytoprotective outcomes. Red and near-infrared light (590, 660 and 850 nm) are absorbed by the metal centres of cytochrome c oxidase (CuA, CuB, heme a and heme a_3_), driving nitric oxide photodissociation and reactivation of complex IV. The resulting bioenergetic recovery (↑ ATP, restored ΔΨm), transient mitochondrial ROS burst and rise in Ca^2+^/cAMP secondary messengers converge on key transcriptional hubs: activation of Nrf2/ARE-driven antioxidant defence (HO-1, NQO1, SOD2); suppression of NF-κB and NLRP3 inflammasome signalling, with reduced TNF-α, IL-1β and IL-6 output; downregulation of the HIF-1α/VEGF angiogenic axis; and engagement of the PGC-1α/AMPK/SIRT1 biogenesis programme (TFAM, NRF1). The integrated molecular response yields antioxidant defence, anti-apoptotic remodelling (↑ Bcl-2/Bax ratio, ↓ caspase-3 activation) and durable cellular survival of photoreceptors and retinal pigment epithelium through coordinated mitochondrial biogenesis and mitophagy.

Under physiological stress, mitochondrial nitric oxide (NO), generated by mitochondrial nitric oxide synthase (mtNOS) and by diffusion of cytosolic NO, competitively binds to the CuB–heme a_3_ binuclear centre. NO competes with O_2_ for the active site, forming a nitrosylated CcO complex (CcO–NO) that is catalytically inactive and that constitutes one of the most physiologically relevant mechanisms of respiratory inhibition. Photon absorption at 660 nm and at NIR wavelengths is currently understood to drive photodissociation of NO from the binuclear centre, regenerating the oxygen-binding configuration and restoring electron flux through complex IV [3,4,15]. The reactivated enzyme increases proton translocation across the inner mitochondrial membrane, raises the proton-motive force (Δp), restores mitochondrial membrane potential (ΔΨm) and consequently enhances ATP synthesis by complex V (F_1_F_0_-ATP synthase) [3,4,15,16,17]. This sequence—photon absorption → NO photodissociation → CcO reactivation → restoration of Δp/ΔΨm → ATP increase—is the molecular cornerstone of PBM in the retina and provides the mechanistic basis for the downstream signalling events described in the following subsections (Figure 1).

In addition to NO photodissociation, two complementary photochemical models have been proposed to account for PBM effects at CcO. First, photoexcitation may directly alter the redox state of the metal centres, transiently increasing the proportion of oxidized CuA available to accept electrons from cytochrome c [15]. Second, photons may interact with structured water layers at the enzyme–lipid interface, modulating proton accessibility to the proton-pumping channels (D- and K-channels) of CcO [4,15]. While these proposals remain partially overlapping and the relative contribution of each mechanism in vivo is debated, all converge on a common phenotypic outcome: an increase in CcO turnover and a measurable rise in mitochondrial ATP output.

### 2.2. Mitochondrial Bioenergetics, Biogenesis and Dynamics

The functional consequence of CcO photoactivation is a measurable increase in mitochondrial ATP production, which has been documented across multiple experimental systems. In aged Drosophila melanogaster, 670 nm exposure increases head ATP content, extends lifespan and improves mobility [11,18]. In a recent controlled wavelength-comparison study in w^1118^ Drosophila with light-induced photoreceptor degeneration, 850 and 950 nm treatments nearly doubled head ATP relative to baseline-recovery flies, while 670, 750, 810 and 850 nm—but not 950 nm—produced significant electrophysiological recovery [18]. These data confirm that PBM-induced bioenergetic gains translate into functional photoreceptor recovery and underscore the importance of wavelength selection.

Beyond the acute bioenergetic boost, repeated PBM exposure engages a transcriptional programme of mitochondrial biogenesis. The master coactivator PGC-1α (peroxisome proliferator-activated receptor gamma coactivator 1-alpha) is upregulated in retinal and RPE models following NIR exposure and, in turn, activates the nuclear respiratory factors NRF1 and NRF2 (here used in the bioenergetic sense, distinct from the Nrf2/Keap1 antioxidant pathway addressed in Section 2.4). NRF1/NRF2 drive transcription of nuclear-encoded mitochondrial proteins and TFAM (mitochondrial transcription factor A), the latter being essential for the replication and transcription of mitochondrial DNA (mtDNA) [12,17,19]. The net result is increased mitochondrial mass, restored COX subunit expression and a renewed capacity for oxidative phosphorylation.

Mitochondrial quality is maintained not only by biogenesis but by a tightly regulated balance between fission and fusion, controlled by the dynamin-related GTPases Drp1 (fission) and Mfn1/Mfn2 plus Opa1 (fusion). Chronic oxidative and metabolic stress in aged retina and in dry AMD models shifts this balance toward fragmentation, with an accumulation of damaged mitochondria that escape clearance. PBM appears to normalize this fission/fusion balance, partly through PGC-1α and partly through restoration of ΔΨm, which is itself a key signal for fusion competence. It also facilitates the selective elimination of dysfunctional mitochondria via PINK1/Parkin-mediated mitophagy [1,12,17]. By coupling biogenesis with mitophagy, PBM supports the replacement of damaged organelles by competent ones. This is particularly relevant to the post-mitotic photoreceptors and the long-lived RPE, where mitochondrial turnover is otherwise limited.

In the mammalian retina, NIR-induced gains in mitochondrial output are paralleled by the recovery of ΔΨm, increased CcO subunit expression and reduced markers of cellular senescence in the RPE and photoreceptors [1,17]. Because the RPE depends on robust ATP availability to perform phagocytosis of photoreceptor outer-segment discs, recycle visual-cycle intermediates and maintain ion transport functions, even modest restoration of mitochondrial output may have disproportionate downstream effects on retinal homeostasis. The convergence of acute (NO photodissociation, ΔΨm restoration), subacute (Ca^2+^/cAMP-dependent signalling; see Section 2.3) and chronic (PGC-1α/TFAM-driven biogenesis) molecular outcomes provides a coherent framework for the durability of PBM benefits observed in clinical trials.

### 2.3. Secondary Messenger Signalling and Convergent Kinase Pathways

CcO photoactivation does not act in isolation: the resulting changes in ΔΨm, the ATP/ADP ratio, redox tone and mitochondrial Ca^2+^ handling propagate to a network of secondary messengers that translate the photonic stimulus into cytoprotective transcriptional programmes [3,4,17]. Restored ΔΨm enhances mitochondrial Ca^2+^ uptake through the mitochondrial calcium uniporter (MCU), which in turn shapes cytosolic Ca^2+^ transients and activates Ca^2+^-dependent enzymes including CaMKII and the calcium-dependent isoforms of adenylate cyclase. The resulting elevation in intracellular cAMP activates protein kinase A (PKA) and the exchange protein activated by cAMP (EPAC), with downstream phosphorylation of CREB and induction of cytoprotective and neurotrophic gene programmes such as BDNF and BCL2 [3,17].

A second convergent node is the phosphoinositide 3-kinase (PI3K)/Akt axis, which is robustly activated by PBM in retinal cell models. Akt phosphorylation inhibits the pro-apoptotic Bcl-2 family member BAD, stabilizes mitochondrial integrity and engages mTORC1 to support protein synthesis and cellular repair. Parallel activation of the mitogen-activated protein kinase (MAPK)/ERK1/2 cascade further reinforces survival signalling, while transient activation of p38 MAPK appears to operate as a stress-adaptive node rather than an apoptotic node at therapeutic PBM doses [3,4,17,19].

Acting in opposition to mTOR, AMP-activated protein kinase (AMPK) responds to subtle, transient drops in the ATP/AMP ratio that precede the steady-state bioenergetic rise. AMPK phosphorylates PGC-1α and supports the biogenesis programme described above, links cellular energetics to autophagy via ULK1, and modulates the activity of the NAD^+^-dependent deacetylase SIRT1. The AMPK–SIRT1–PGC-1α axis is therefore a critical molecular hub integrating PBM-induced bioenergetic signals with mitochondrial biogenesis, antioxidant defence and circadian regulation (Section 2.7) [3,12,17].

### 2.4. Redox Signalling and the Nrf2/Keap1/ARE Antioxidant Programme

Reactive oxygen species (ROS) are unavoidable by-products of cellular respiration and accumulate in photoreceptors and the RPE with ageing and in genetic retinal dystrophies. Excessive ROS damages mitochondrial DNA, lipids and proteins, perpetuating bioenergetic failure and apoptotic signalling [1,2]. PBM exerts a biphasic, dose-dependent effect on ROS: at therapeutic fluences, photon delivery produces a transient and spatially confined ROS burst—predominantly mitochondrial H_2_O_2_—that functions as a redox second messenger rather than as a damaging agent [3,17,19].

The central molecular target of this redox signal is the Kelch-like ECH-associated protein 1 (Keap1)/nuclear factor erythroid 2-related factor 2 (Nrf2)/antioxidant response element (ARE) pathway. Under basal conditions, Nrf2 is sequestered in the cytoplasm by Keap1, which targets it for Cullin-3-mediated ubiquitination and proteasomal degradation. Oxidation of redox-sensitive cysteine residues on Keap1 (notably Cys151, Cys273 and Cys288) disrupts this complex, allowing Nrf2 to translocate to the nucleus, heterodimerize with small Maf proteins and bind ARE sequences in the promoters of cytoprotective genes. The Nrf2-driven transcriptional programme induced by PBM includes heme oxygenase-1 (HO-1), NAD(P)H quinone dehydrogenase 1 (NQO1), the glutamate–cysteine ligase catalytic and modifier subunits (GCLC/GCLM, rate-limiting for glutathione biosynthesis), thioredoxin reductase 1 (TXNRD1), superoxide dismutase 2 (SOD2), catalase and glutathione peroxidase (GPx) [3,17,19]. Collectively, these enzymes neutralize the chronic, pathogenic ROS burden of an aged or diseased retina while preserving the physiological redox signalling required for cellular homeostasis.

In RPE cultures exposed to oxidative or hypoxic stress, multiwavelength PBM consistently suppresses pathogenic ROS generation, increases reduced/oxidized glutathione (GSH/GSSG) ratios and confers cytoprotection in an Nrf2-dependent manner [19]. The integration of PBM with the Nrf2/Keap1/ARE axis provides one of the strongest molecular rationales for its disease-modifying effect in dry AMD, where oxidative damage of the RPE is a defining pathogenic event, and supports its mechanistic plausibility in RP and CSCR, in which redox dysregulation also contributes to outer-retinal injury.

### 2.5. Anti-Apoptotic Remodelling and Cytoprotection

Mitochondrial apoptosis, triggered by permeabilization of the outer mitochondrial membrane and cytochrome c release into the cytosol, is a final common pathway of photoreceptor and RPE death in AMD, RP and chronic CSCR [1,2,20]. The balance between pro-apoptotic (Bax, Bak, Bid, BAD) and anti-apoptotic (Bcl-2, Bcl-xL, Mcl-1) members of the Bcl-2 family at the outer mitochondrial membrane is therefore a critical molecular determinant of retinal cell survival.

PBM consistently shifts this balance toward survival. Across retinal cell models, NIR exposure increases the Bcl-2/Bax ratio, reduces cytosolic cytochrome c, attenuates activation of the apoptotic initiator caspase-9 and the executioner caspase-3, and limits PARP cleavage [3,17,19]. These effects are mechanistically linked to upstream events described in previous sections: restored ΔΨm stabilizes the outer mitochondrial membrane and reduces susceptibility to mitochondrial permeability transition pore (mPTP) opening; Akt-mediated phosphorylation of BAD sequesters this pro-apoptotic factor away from Bcl-xL; and Nrf2-driven antioxidant defence limits ROS-induced oxidation of cardiolipin, a critical step in cytochrome c release. In parallel, PBM upregulates the brain-derived neurotrophic factor (BDNF)/TrkB and ciliary neurotrophic factor (CNTF) axes in retinal models, providing additional cytoprotective input independent of mitochondrial signalling [3,4,17].

A complementary cytoprotective axis is the regulation of autophagy and selective mitophagy. PINK1 (PTEN-induced kinase 1) accumulates on the outer membrane of depolarized mitochondria, recruits the E3 ubiquitin ligase Parkin and ubiquitinates outer-membrane substrates such as Mfn1/2 and Miro, marking damaged organelles for lysosomal degradation. By transiently lowering ΔΨm in compromised mitochondria while restoring it in functional mitochondria, PBM may favour preferential clearance of damaged organelles through PINK1/Parkin-mediated mitophagy, complementing the PGC-1α-driven biogenesis described in Section 2.2 [12,17].

### 2.6. Anti-Inflammatory and Anti-Angiogenic Signalling: NF-κB, NLRP3 and the HIF-1α/VEGF Axis

PBM modulates several signalling cascades implicated in retinal pathology. At the molecular level, photoactivation of CcO and the resulting redox and Ca^2+^ shifts attenuate activation of the nuclear factor κB (NF-κB) pathway. Under inflammatory stress, IκB kinase (IKK) phosphorylates IκBα, leading to its proteasomal degradation and freeing the p65/p50 NF-κB heterodimer to translocate to the nucleus and drive transcription of pro-inflammatory cytokines including TNF-α, IL-1β, IL-6 and IL-8, chemokines such as MCP-1 and IL-8, and matrix metalloproteinases (MMP-2 and MMP-9). PBM stabilizes IκBα, reduces nuclear p65 translocation and consequently lowers cytokine, chemokine and MMP output in RPE and microglial cell models [3,4,17,19].

A parallel and increasingly recognized target is the NLRP3 inflammasome, a cytosolic multiprotein complex composed of the sensor NLRP3, the adaptor ASC and procaspase-1. Activation of NLRP3 in RPE and microglia, driven by oxidized lipids, complement fragments (C3a/C5a) and lysosomal destabilization, leads to maturation of IL-1β and IL-18 and, in advanced settings, to gasdermin-D-mediated pyroptosis. PBM reduces NLRP3 priming and assembly, partly through Nrf2-mediated suppression of ROS-driven signal 2 and partly through restoration of mitochondrial homeostasis, which is itself a major upstream regulator of inflammasome activity [3,17,19].

PBM also attenuates the hypoxia-inducible factor 1-alpha (HIF-1α)/vascular endothelial growth factor (VEGF) axis. Under hypoxic or pseudo-hypoxic conditions, stabilization of HIF-1α drives transcription of VEGFA and other angiogenic and inflammatory targets relevant to AMD, CSCR and other diseases characterized by abnormal vascular permeability and choroidal dysfunction. By restoring oxidative phosphorylation efficiency and reducing the pseudo-hypoxic mitochondrial dysfunction characteristic of an aged RPE, PBM lowers HIF-1α stabilization and consequently dampens VEGFA transcription. The 590 nm component of multiwavelength PBM appears to be particularly active on VEGF expression and RPE pump function in cell culture, providing a molecular rationale for the clinical observation that 590/660/850 nm exposure reduces drusen volume growth and the incidence of geographic atrophy in dry AMD [4,5,6,21].

In AMD models, 670 nm light has been shown to reduce retinal inflammation and to upregulate CcO expression and activity, with parallel suppression of microglial activation markers [22]. Multiwavelength PBM (590/660/850 nm) consistently reduces drusen volume growth and the incidence of geographic atrophy in dry AMD, consistent with combined anti-inflammatory, anti-angiogenic and pro-metabolic effects acting through the NF-κB, NLRP3 and HIF-1α/VEGF nodes described above [5,6,22].

### 2.7. Molecular Crosstalk Between PBM Signalling and the Circadian Machinery

A defining feature of mitochondrial biology in the retina is its tight integration with the circadian molecular clock. The core mammalian clock comprises the transcriptional activators BMAL1 (ARNTL) and CLOCK, which form heterodimers that drive expression of the repressors PER1–3 and CRY1–2; the latter, in turn, inhibit BMAL1/CLOCK to close an approximately 24 h transcriptional feedback loop. Additional stabilizing loops involve the nuclear receptors REV-ERBα/β and RORα/β, which competitively regulate BMAL1 transcription [12,20]. These factors are robustly expressed in retinal neurons, RPE and Müller cells, and govern daily oscillations in photoreceptor outer-segment shedding, RPE phagocytosis, visual-cycle activity, choroidal blood flow and intraocular pressure.

Critically for PBM, the molecular clock directly regulates mitochondrial function. BMAL1/CLOCK targets include PGC-1α and several nuclear-encoded ETC subunits, and REV-ERBα represses mitochondrial biogenesis. The NAD^+^-dependent deacetylases SIRT1 (nuclear) and SIRT3 (mitochondrial) couple cellular redox state to clock output: NAD^+^ levels themselves oscillate diurnally, peaking in the early morning, and SIRT1/SIRT3 in turn deacetylate BMAL1, PGC-1α and key mitochondrial enzymes (SOD2, IDH2, complex I subunits) to align bioenergetic capacity with the active phase of the cycle [12,17,20]. AMPK, addressed in Section 2.3, also phosphorylates CRY1 and destabilizes it, providing an additional bidirectional link between energy status and clock phase.

Because CcO activity itself follows a circadian rhythm and because the molecular substrates of PBM (mitochondrial ROS, ATP, NAD^+^/SIRT1, PGC-1α) are clock-controlled, the response to PBM is not constant across the day. The mitochondrial state during the morning peak—high NAD^+^, deacetylated PGC-1α, low REV-ERBα-mediated repression—is bioenergetically primed to amplify the photonic signal. In contrast, in the afternoon and evening, BMAL1-driven transcription has waned, SIRT1 activity is lower and the same photon dose finds a substrate that is poised for downregulation rather than amplification, providing a molecular rationale for the time-dependent efficacy data reviewed in Section 3 [11,12,13,14,20]. Conversely, PBM-induced changes in cellular redox tone and NAD^+^ availability may themselves feed back onto the SIRT1/BMAL1 axis, suggesting a bidirectional relationship in which PBM not only follows but also tunes the molecular clock—a hypothesis that, although not yet formally tested in the retina, is an attractive avenue for future mechanistic studies.

The molecular basis of this circadian dependence can be traced to a defined chain of events linking the core clock to CcO activity. During the active phase, the BMAL1/CLOCK heterodimer drives transcription of genes that govern oxidative metabolism. These include PGC-1α and NAMPT, the rate-limiting enzyme of NAD^+^ salvage. Clock-driven NAMPT expression makes intracellular NAD^+^ oscillate, peaking in the early morning. This peak sets the activity ceiling for the NAD^+^-dependent deacetylases SIRT1 and SIRT3. Nuclear SIRT1 then activates BMAL1 and PGC-1α, reinforcing transcription of electron transport chain subunits and TFAM. In parallel, mitochondrial SIRT3 activates matrix enzymes, including complex I subunits, IDH2, the antioxidant SOD2 and components of the CcO assembly and catalytic machinery. Through this BMAL1/CLOCK → NAMPT → NAD^+^ → SIRT1/SIRT3 cascade, the abundance and catalytic efficiency of CcO fall under direct circadian control, so that complex IV turnover and ATP output are intrinsically highest in the morning. The repressive limb of the clock acts reciprocally. Accumulating PER/CRY closes the feedback loop and lowers BMAL1/CLOCK output later in the day, REV-ERBα represses PGC-1α and the biogenesis programme, and AMPK-mediated phosphorylation destabilizes CRY1. Against this oscillating substrate, a fixed photon dose is amplified in the morning, when NAD^+^, deacetylated PGC-1α and SIRT3-activated complex IV are all maximal. The same dose meets a repression-biased system in the afternoon and evening, when REV-ERBα activity is rising and SIRT activity is falling. This sequence provides the theoretical basis for chronotherapeutic PBM. It also predicts that raising NAD^+^ availability or reinforcing BMAL1/SIRT signalling could widen the temporal window of responsiveness, a prediction that is testable in retinal models [11,12,13,14,17,20] (Table 1).

### 2.8. Wavelength Specificity and Dose Dependence: From Photophysics to Phenotype

Different wavelengths produce distinct biological outputs, even when delivered at equal photon counts. A direct comparison in light-damaged Drosophila showed that 670, 750, 810 and 850 nm improved electroretinographic responses and photoreceptor visibility, with 850 nm yielding the highest effect; 950 nm, in contrast, failed to restore photoreceptor function despite increasing ATP, suggesting a dissociation between bioenergetic and electrophysiological recovery at the extreme NIR end [18]. A 590 nm control treatment did not reproduce the recovery, ruling out a non-specific effect via metarhodopsin reconversion and reinforcing the CcO-mediated mechanism [18].

In clinical practice, the Valeda multiwavelength platform combines yellow (590 nm), red (660 nm) and NIR (850 nm) light, with each wavelength engaging distinct molecular nodes: 590 nm acts predominantly on HIF-1α/VEGF, NF-κB-driven cytokine production and RPE Na^+^/K^+^-ATPase pump function; 660 nm interacts with the CuB centre of CcO, enhancing O_2_ binding and driving the acute bioenergetic response; and 850 nm is absorbed at the CuA centre, supporting electron entry into complex IV and sustaining anti-inflammatory and biogenesis-related signalling [6,23]. Dose, fluence and treatment cadence remain critical parameters: too low a dose may be sub-therapeutic, whereas excessive fluence can exhaust the photon–chromophore response and tip the redox balance from signalling to damage, in line with the biphasic dose–response characteristic of PBM and consistent with the Arndt–Schulz law [3,4].

It is important to emphasize that this dose dependence reflects a genuinely dual, context-sensitive biology rather than a uniformly protective effect, and that several of the molecular axes engaged by PBM can be modulated in opposite directions depending on irradiation parameters and the redox state of the target tissue. The transient mitochondrial ROS burst that underlies adaptive Nrf2/ARE signalling at therapeutic fluences (Section 2.4) is the same chemistry that, when the photon dose is sub-therapeutic or excessive, can tip the system toward aberrant ROS overaccumulation, depletion of reduced glutathione, oxidation of mitochondrial DNA and lipids, and frank cellular injury rather than cytoprotection. The biphasic curve therefore has two failure modes: insufficient stimulation that does not reach the threshold for CcO reactivation, and overstimulation in which sustained oxidant generation overwhelms antioxidant buffering and converts a redox signal into oxidative stress. Consistent with this, the regulation of the NF-κB inflammatory cascade and the HIF-1α/VEGF angiogenic axis by PBM is not uniform across the literature: while most retinal and RPE studies report stabilization of IκBα with reduced NF-κB nuclear translocation and lower VEGF output, other reports describe transient NF-κB activation or unchanged or even increased VEGF expression under certain conditions. Several confounders plausibly underlie this phenotypic heterogeneity, including differences in wavelength and combinations thereof, irradiance and total fluence, pulsed versus continuous delivery, the number and spacing of treatment sessions, the baseline oxidative and inflammatory tone of the model (healthy versus stressed, normoxic versus hypoxic), the cell type and species studied, and inconsistent or incomplete reporting of dosimetric parameters that precludes direct cross-study comparison. A balanced reading of the evidence thus indicates that PBM should be regarded as a bidirectional modulator whose anti-inflammatory and anti-angiogenic benefits are conditional on operating within a defined therapeutic window, and that discordant outcomes across studies are best interpreted as reflecting departures from that window and differences in experimental context rather than true contradictions in the underlying biology [3,4,17,19].

### 2.9. Tissue Penetration and Optical Window: Rationale for Closed-Eye, Transpalpebral Delivery

An additional biophysical determinant of PBM efficacy in the posterior segment is the tissue penetration profile of red and near-infrared light. Within the so-called “optical window” of biological tissues (approximately 600–1100 nm), absorption by the main endogenous chromophores—haemoglobin, melanin and water—reaches a relative minimum, while photon scattering, although significant, still permits substantial forward transmission [3,4,15]. Photons in this range therefore traverse skin, subcutaneous tissue and the eyelid with comparatively low attenuation, whereas wavelengths below ~600 nm (including violet, blue, green and yellow light) are strongly absorbed by superficial chromophores and dissipate within the first few hundred micrometres of tissue. Red light at 660–670 nm and NIR light at 810–850 nm have repeatedly been shown to deliver biologically meaningful fluences to deep targets, including the brain, peripheral nerves and skeletal muscle, when applied transcutaneously [3,4]. Although the closed eyelid attenuates incident photons by roughly one order of magnitude in the red and NIR ranges, the residual fluence that reaches the retina remains within the therapeutic window required to photoactivate CcO at the photoreceptor and RPE level [4,6,15].

This transmission profile provides the biophysical rationale for the closed-eye, transpalpebral delivery protocol adopted by multiwavelength PBM in dry AMD and by red-light monotherapy in myopia. With the eyelid closed, 660 and 850 nm photons can still reach the outer retina at fluences sufficient to engage CcO-mediated signalling (Section 2.1), while the cornea, lens and anterior segment are shielded from direct exposure and the patient is protected from a startle response or accidental glare. Closed-eye delivery also obviates the need for pupil dilation, simplifies treatment logistics and removes the risk of photochemical or thermal injury to the anterior segment that could arise from direct, undiffused illumination. In contrast, 590 nm yellow light is more strongly absorbed by the eyelid skin and haemoglobin in the palpebral microvasculature, but is delivered in the multiwavelength regimen to act primarily on the superficial RPE and choroidal targets, where modest transmission is sufficient to modulate the HIF-1α/VEGF, NF-κB and Na^+^/K^+^-ATPase pathways described in Section 2.4 and Section 2.6 [4,6,23]. Importantly, closed-eye delivery shifts the role of the eyelid from a passive obstacle to a built-in optical diffuser and dose attenuator, which contributes to the homogeneous, sub-erythemic and non-thermal exposure documented across LIGHTSITE I–III and IIIB and to the favourable safety profile of the device-based platforms currently in clinical use [4,5,6,24,25,26,27].

## 3. Circadian Mitochondrial Rhythms and the Timing of PBM

### 3.1. Diurnal Oscillation of Mitochondrial Function

Mitochondrial respiration, ATP synthesis and the production of metabolic by-products are not constant across the 24 h cycle but show robust circadian oscillations [12,20]. In Drosophila, mitochondrial ATP production peaks during the early morning hours, declines during the day and reaches its nadir at night [13,20]. Comparable rhythms have been documented in mammalian tissues, including the retina and RPE, and are governed by the molecular clock and clock-regulated changes in mitochondrial dynamics, biogenesis, fission/fusion balance and metabolite supply, as detailed at the molecular level in Section 2.7 [12].

These oscillations are biologically meaningful for the retina, where photoreceptor outer-segment shedding, RPE phagocytosis and visual-cycle activity all exhibit daily rhythms. Ageing and chronic disease blunt circadian amplitude—a phenomenon often referred to as “circadian disruption” or “clock dampening”—contributing to mitochondrial inefficiency and the metabolic vulnerability of the outer retina [1,12].

### 3.2. Time-Dependent Efficacy of NIR Exposure

Because CcO activity and its molecular cofactors (NAD^+^, SIRT1/3, PGC-1α) follow a circadian rhythm, the response to PBM is not constant across the day. Preclinical work in Drosophila has demonstrated that 670 nm exposure significantly improves mitochondrial function only when administered during a narrow morning window (approximately 08:00–11:00); the same exposure delivered later in the day produces little or no benefit [13,14,20].

In humans, a controlled exposure study in healthy individuals (34–70 years of age) reported a mean ≈ 17% improvement in colour contrast sensitivity along the protan and tritan axes after a single 3 min exposure to 670 nm deep red light delivered between 08:00 and 09:00. The benefit persisted for at least one week, with up to 20% improvement in older participants. When the same protocol was later repeated between 12:00 and 13:00 in a subgroup of participants, no improvement was observed, supporting a time-restricted window for cone-mediated photic benefit [11,14]. These early human data are intriguing and suggest that the temporal dimension of PBM dosing may be an important variable. They derive, however, from a small study of a surrogate functional endpoint in healthy participants and should be interpreted as preliminary; whether timing influences clinical outcomes in retinal disease, and how its importance compares with that of wavelength and fluence, remains to be established.

### 3.3. Implications for Chronotherapeutic PBM

From a mechanistic standpoint, the rationale for morning delivery is biologically plausible: photon-driven reactivation of CcO is expected to be most productive when mitochondria are already biased toward high respiratory activity, when NAD^+^ availability and SIRT1/3 activity peak, and when BMAL1-driven transcription of PGC-1α and ETC subunits is at its maximum (Section 2.7). Delivering PBM during the afternoon, when mitochondrial activity is naturally declining and REV-ERBα-mediated repression is rising, may be analogous to attempting to amplify a signal that is already being downregulated by the cellular environment [11,12,13,14].

Translating this principle into clinical practice raises several open questions. First, the optimal window is likely to vary across patients depending on chronotype, age and disease stage; older individuals and those with advanced retinal degeneration may have phase-shifted or dampened rhythms that require individualized scheduling. Second, the chronic, repeat-dosing regimens used in LIGHTSITE and other clinical programmes have so far not formally stratified by time of day; retrospective analyses and prospective chronotherapeutic trials are warranted. Third, integration with environmental cues (light exposure, sleep, meal timing) that synchronize the molecular clock may enhance and stabilize PBM responses [12,20]. Recognizing PBM as a chronotherapy provides a conceptually new axis for protocol optimization, complementing the more traditional parameters of wavelength, fluence and treatment frequency. It should be emphasized, however, that the superiority of morning delivery currently rests on a limited body of preclinical work and a single small human study using a surrogate endpoint. At present it is best regarded as a biologically grounded, emerging hypothesis that requires confirmation in adequately powered, time-stratified clinical trials before it can be adopted as a treatment rule; the existing clinical PBM evidence summarized below was generated without controlling for time of day.

## 4. Clinical Applications in Retinal Diseases

Before reviewing the clinical evidence disease by disease, it is useful to map the molecular actions of PBM detailed in Section 2 onto the hallmark pathological alterations of each indication, since the therapeutic value of multiwavelength PBM derives from the correspondence between specific molecular nodes and specific lesions rather than from a generic protective effect. RPE metabolic dysfunction, the defining lesion of dry AMD, combines bioenergetic failure, lipofuscin and drusen accumulation, chronic oxidative damage and pseudo-hypoxic NLRP3 and complement activation; here, PBM acts through CcO reactivation and PGC-1α/TFAM-driven biogenesis to restore the ATP supply required for outer-segment phagocytosis and Na^+^/K^+^-ATPase pumping, through the Nrf2/Keap1/ARE axis to counter the oxidative burden, and through NF-κB and NLRP3 suppression to dampen the parainflammation that drives progression to geographic atrophy. Photoreceptor degenerative loss, the central lesion of retinitis pigmentosa and a downstream feature of advanced AMD, reflects mutation-driven or stress-driven mitochondrial collapse converging on cytochrome c release and caspase activation; PBM addresses this node through ΔΨm restoration, anti-apoptotic remodelling of the Bcl-2/Bax ratio and neurotrophic BDNF/CNTF support, with the important caveat that a residual population of viable photoreceptors retaining functional CcO is required for any response. Choroidal microvascular dysregulation, prominent in central serous chorioretinopathy and in the pachychoroid spectrum, couples impaired RPE pump function and fluid mishandling to HIF-1α/VEGF-driven vascular hyperpermeability; the 590 nm component of multiwavelength PBM is mechanistically matched to this lesion, acting on RPE Na^+^/K^+^-ATPase activity and on the HIF-1α/VEGF and NF-κB axes to restore the outer blood–retinal barrier. In pathological myopia, the relevant target is not degeneration but aberrant axial growth, and the molecular correlate of red light therapy is light-dependent retinal dopamine release together with scleral and choroidal metabolic modulation. Framing the indications in this way clarifies that multiwavelength PBM is not uniformly applicable but exhibits a degree of pathological adaptability and therapeutic specificity: the same photonic platform engages different dominant molecular nodes according to the predominant lesion, which in turn predicts where benefit is most likely and where viable target tissue may be insufficient for a response.

### 4.1. Age-Related Macular Degeneration

Dry AMD is the most extensively investigated retinal indication for PBM and is currently the only FDA-authorized indication. The LIGHTSITE clinical programme has progressively built evidence for multiwavelength PBM with the Valeda Light Delivery System (590/660/850 nm). LIGHTSITE I, a single-centre randomized sham-controlled study, demonstrated improvements in BCVA, contrast sensitivity and central drusen volume after a series of nine treatments delivered over 3–5 weeks [24]. LIGHTSITE II, a multicentre randomized trial, confirmed a statistically significant BCVA gain of approximately four letters at 9 months in PBM-treated eyes versus 0.5 letters in sham, with stable drusen volume and reduced geographic atrophy growth [25].

LIGHTSITE III, the pivotal randomized double-masked controlled trial, enrolled 100 subjects (148 eyes) with intermediate dry AMD and BCVA between 20/32 and 20/100. Subjects received multiwavelength PBM or sham every four months, with a series of nine treatments per cycle, over 24 months. At month 13, the PBM group showed a mean BCVA gain of 5.4 ETDRS letters and a significant reduction in new-onset geographic atrophy (odds ratio 9.4; *p* = 0.024) [5]. At month 24, the BCVA benefit was sustained (mean 5.9 letters; *p* = 0.0015), with 6.8% of PBM eyes progressing to incident GA versus 24% in the sham group (approximately 73% relative reduction; OR 4.2; *p* = 0.007), alongside reduced drusen volume growth and a favourable safety profile, with no signs of phototoxicity [5,26]. The molecular substrate of this disease-modifying effect is consistent with the converging actions of Nrf2-driven antioxidant defence, NLRP3 inflammasome suppression, HIF-1α/VEGF dampening and PGC-1α-mediated mitochondrial biogenesis in the RPE described in Section 2.

The open-label LIGHTSITE IIIB extension trial followed subjects from LIGHTSITE III who resumed PBM after a ~20-month interruption and received additional treatment series over 13 months. Patients who had previously received active PBM regained approximately five BCVA letters upon retreatment, and more than 60% of PBM-treated subjects continued to show a >1-line vision benefit, with extended benefit reported up to 4.5 years from initial therapy [27]. Subjects originally randomized to sham who were switched to active PBM in the extension showed stabilization of vision. These data support PBM as a disease-modifying, repeat-dosed therapy with durable effects, particularly when initiated in early-to-intermediate disease [27].

### 4.2. Retinitis Pigmentosa

Retinitis pigmentosa (RP) comprises a heterogeneous group of inherited retinal dystrophies, with over 40 genes implicated in non-syndromic forms and more than 50 in syndromic forms. Beyond the limited subset of patients eligible for RPE65-targeted gene therapy (voretigene neparvovec), there is no curative treatment, and mitochondrial dysfunction and oxidative stress are common downstream pathogenic features shared across genotypes [9,21]. This convergent biology—in which mutation-specific primary defects feed into a common bioenergetic and redox failure of the photoreceptor–RPE complex—provides a plausible rationale for PBM as a genotype-agnostic neuroprotective strategy. In contrast to dry AMD, however, the clinical evidence in RP is at an early stage, limited to small, short-term studies without randomized or sham-controlled comparison; so, the following findings should be read as preliminary and hypothesis-generating.

In a prospective short-term study, 12 RP patients (24 eyes) underwent nine sessions of multiwavelength PBM with the Valeda Light Delivery System (590 nm at 4 mW/cm^2^; 660 nm at 65 mW/cm^2^; 850 nm at 0.6 mW/cm^2^), delivered three times per week on alternate days over three weeks, with evaluations four weeks after the final session [9]. LogMAR BCVA improved significantly from a mean of 0.62 to 0.53 (*p* = 0.001). Mean deviation on fundus-automated perimetry (Compass) improved from −19.87 dB to −19.45 dB, with parallel positive trends in pattern standard deviation and fundus perimetry defect index (non-significant at this short follow-up). No adverse events and no abnormalities on OCT or ERG were observed [9]. These early findings are encouraging but are limited by the small sample, the short follow-up and the absence of a control arm, and should be regarded as preliminary. Any benefit would presumably depend on a residual population of viable cells with functional CcO remaining to respond to the photonic stimulus.

### 4.3. Central Serous Chorioretinopathy

Central serous chorioretinopathy (CSCR) is characterized by serous detachment of the RPE and neurosensory retina, with choroidal vascular dysregulation and impaired RPE pump function. While acute episodes often resolve spontaneously, chronic CSCR may lead to irreversible structural and functional damage, and current treatment options (photodynamic therapy, subthreshold micropulse laser, mineralocorticoid antagonists) have variable efficacy and limitations [7,8,28]. The evidence for PBM in CSCR is currently the most preliminary of the indications reviewed here, consisting of isolated case reports rather than controlled series; the observations below are therefore anecdotal and intended only to motivate formal study.

Two recent reports describe rapid and durable responses to PBM in CSCR. In a prospective case of acute CSCR treated with multiwavelength PBM (Valeda, 590/660/850 nm), nine treatment sessions over 3–5 weeks led to a reduction in central retinal thickness from 544 to 367 µm after the first three sessions and to complete fluid resolution by the end of the series, with BCVA improving from 55 to 80 ETDRS letters; the benefit was maintained over one year with a single repeat series at six months [7]. In a separate case of chronic CSCR with a giant serous pigment epithelium detachment refractory to eplerenone, dual-wavelength low-level light therapy (590 nm and 625/630 nm) administered weekly for four weeks and then bi-weekly for two months resulted in progressive flattening of the PED, complete subretinalfluid resorption by six months, BCVA improvement from 20/80 to 20/25 and microperimetry-confirmed mean macular sensitivity rising from 7.4 dB to 26.5 dB at 12 months [8].

Mechanistically, a benefit of PBM in CSCR would be plausible within the molecular framework outlined in Section 2, through restoration of RPE Na^+^/K^+^-ATPase pump activity by ATP repletion, suppression of HIF-1α/VEGF and inflammatory mediators by the 590 nm component, and bioenergetic support of choroidal–RPE interactions via CcO activation [7,8,19,23]. These single-case observations cannot, however, establish causality, and at most they motivate controlled prospective studies of PBM as a possible non-invasive option in CSCR, particularly given the global verteporfin shortage.

### 4.4. Pathological Myopia

Childhood and adolescent myopia is rising rapidly in prevalence and is associated with long-term risks of myopic maculopathy, glaucoma and retinal detachment. Repeated low-level red light (RLRL) therapy, typically delivered at 650 nm via semiconductor laser-based home devices, has emerged as a non-invasive strategy to slow axial elongation and refractive progression [10,29]. Of the non-AMD indications, myopia has the broadest controlled evidence base, including randomized trials of RLRL; nonetheless this application differs mechanistically from the others (growth control rather than neuroprotection), questions of durability and rebound after cessation remain unresolved, and the data should still be regarded as developing rather than definitive.

In a six-month, single-arm, single-centre study of 32 adolescents (mean age 11.5 years) with mild-to-moderate myopia, RLRL therapy delivered twice daily for three minutes (650 ± 10 nm; ≈1.07–1.43 mW/cm^2^ at 10 cm; ≈1600 lux) was associated with stabilization of axial length and a small but statistically significant improvement in spherical equivalent refraction (from −2.39 ± 2.21 D at baseline to −2.01 ± 2.12 D at six months; *p* < 0.05). Secondary measurements—intraocular pressure, corneal parameters, choroidal thickness, foveal thickness and superficial and deep macular vessel density on OCT angiography—remained stable, and no adverse events were reported [10]. These findings replicate previous randomized data showing 0.06–0.70 D improvements over 6–12 months [29] and importantly extend safety to retinal microvasculature.

The proposed molecular substrate involves light-dependent dopamine signalling in the retina—a key inhibitor of ocular growth, acting predominantly through D1 and D2 receptors on amacrine cells and downstream cAMP/PKA cascades—together with mitochondrial enhancement of scleral and RPE metabolic activity and modulation of the choroidal vasculature [10,30]. As with other PBM applications, rebound after cessation has been described, suggesting that ongoing treatment may be required to maintain benefit and reinforcing the need for long-term controlled trials.

### 4.5. Preclinical Models of Photoreceptor Injury

Preclinical wavelength comparison studies provide some of the strongest mechanistic evidence for PBM in retinal disease. In light-damaged w^1118^ Drosophila, exposure to 670, 750, 810 and 850 nm light for two 30 min periods daily over 10 days produced significant recovery of ERG photoreceptor responses and on-transients, with 850 nm yielding values comparable to those of undamaged controls; 950 nm did not restore electrophysiological function despite raising ATP, and a 590 nm control did not produce recovery, excluding non-specific opsin-cycling effects [18]. Photoreceptor structural integrity, evaluated by cornea neutralization autofluorescence, improved across all NIR groups and was greatest at 810–850 nm [18].

These results are consistent with previous reports that 670 nm protects photoreceptors from light-induced damage and methanol toxicity, increases ATP and CcO expression in aged retinas, and reduces inflammation in murine AMD models [22,31]. Together, the preclinical data provide a coherent mechanistic rationale that is consistent with the clinical observations described above, linking the photophysical events at CcO to the integrated molecular cytoprotective programme detailed in Section 2. They do not, by themselves, demonstrate clinical efficacy, which must be established in controlled trials for each indication.

## 5. Safety and Tolerability

Across preclinical and clinical studies, PBM has shown a consistently favourable safety profile. The LIGHTSITE I–III and IIIB trials reported no signs of phototoxicity over 24 months and beyond, with adverse events comparable to the underlying disease background [5,24,25,26,27]. In RP, CSCR and myopia studies, no significant adverse events, no OCT or ERG abnormalities and no changes in macular retinal blood flow density have been reported [7,8,9,10]. Importantly, multiwavelength PBM is delivered at non-thermal, sub-erythemic doses, with treatment sessions lasting less than five minutes and not requiring pupil dilation [4,6]. The biphasic dose–response of PBM (Section 2.8) underscores the importance of staying within the therapeutic window, where photonic stimulation drives signalling rather than damaging photochemistry. These properties make PBM particularly attractive for paediatric (myopia) and elderly (AMD) populations.

## 6. Limitations and Future Directions

Despite encouraging evidence, important molecular and clinical gaps remain. Across the literature, heterogeneity in irradiance reporting (illuminance, power density, fluence and photon count) complicates direct cross-study comparisons; standardization should follow the recommendations of reporting photon count and fluence alongside conventional metrics [18,32]. Wavelength selection is empirical in most clinical protocols; head-to-head comparisons of single- versus multiwavelength regimens, informed by the differential molecular targets engaged by 590, 660 and 850 nm light (Section 2.8), are needed in human retinal disease.

The clinical evidence base must also be appraised with its methodological constraints clearly in view. Even the strongest dataset, the LIGHTSITE programme in dry AMD, rests on a pivotal trial (LIGHTSITE III) of modest size (100 subjects, 148 eyes) with a 24-month horizon, and the supporting evidence for retinitis pigmentosa, central serous chorioretinopathy and the other indications is confined to small single-arm series, single-centre cohorts and individual case reports. Several recurring limitations therefore qualify the current literature: a limited cohort scale, which constrains statistical power and the detection of subgroup effects; short-to-intermediate follow-up that is insufficient to establish durability of benefit or long-term prognosis in chronic, progressive diseases; a scarcity of multicentre, randomized, sham-controlled verification outside dry AMD, with consequent vulnerability to single-centre and selection bias; and the absence of standardized, structurally validated functional and anatomical endpoints across studies. Compounding these design limitations, the PBM intervention parameters themselves vary widely between independent reports, including wavelength and wavelength combinations, irradiance and total fluence, pulsed versus continuous emission, session duration, the number of sessions per cycle and retreatment intervals, and inconsistent and frequently incomplete dosimetric reporting; this parametric heterogeneity directly undermines the uniformity and reproducibility of outcomes and frustrates meta-analytic synthesis. To move the field toward standardized clinical regimens, future work should (i) adopt and report a minimum dosimetric dataset, expressing dose as photon count and fluence at the retinal plane alongside conventional irradiance and illuminance metrics; (ii) prioritize adequately powered, multicentre, randomized, sham-controlled trials with predefined, structurally validated functional and anatomical endpoints and follow-up extending well beyond 24 months; (iii) converge on harmonized treatment protocols (wavelength set, fluence, cadence and retreatment interval) so that effect sizes can be compared and pooled across centres; (iv) incorporate the chronotherapeutic dimension by prospectively stratifying or controlling for time of day and chronotype; and (v) extend this rigorous, parameter-standardized methodology beyond dry AMD to retinitis pigmentosa, central serous chorioretinopathy and other underserved retinal diseases. Addressing these restrictive factors in a coordinated way is a prerequisite for translating the favourable but still preliminary clinical signals into reproducible, guideline-grade evidence.

From a molecular standpoint, several questions deserve systematic dissection in retinal cell types: the relative contribution of Ca^2+^, cAMP/PKA, PI3K/Akt, MAPK/ERK and AMPK signalling to PBM-induced cytoprotection; the in vivo regulation of the Nrf2/Keap1/ARE axis and NLRP3 inflammasome activity by PBM; the kinetics and reversibility of PGC-1α/TFAM-driven mitochondrial biogenesis after repeated treatment series; the role of PINK1/Parkin-mediated mitophagy in clearing dysfunctional mitochondria from the RPE and photoreceptors; and the genetic interaction of PBM with the molecular clock genes (BMAL1, CLOCK, PER, CRY, REV-ERBα) in the retina, an essentially unexplored area that is particularly attractive given the time-dependent efficacy data. Multi-omics approaches (transcriptomics, proteomics, metabolomics, redox proteomics) applied to retinal organoids, iPSC-derived RPE and animal models exposed to PBM at different times of day will be instrumental in resolving these questions.

Clinically, the next generation of PBM trials should (i) prospectively stratify treatment delivery by time of day and individual chronotype; (ii) define optimal retreatment intervals to prevent loss of benefit, as suggested by LIGHTSITE IIIB and by rebound after RLRL cessation in myopia; (iii) explore combination strategies with anti-VEGF therapy, complement inhibitors and gene-based approaches in selected indications, exploiting molecular synergies at the level of VEGF, complement and bioenergetic pathways; and (iv) extend rigorous controlled evaluation to RP, CSCR and other underserved retinal diseases.

## 7. Conclusions

Photobiomodulation is evolving from an empirical phototherapy into a mechanism-based, molecularly grounded treatment for retinal disease. Photon absorption by cytochrome c oxidase ignites a coordinated molecular programme that encompasses restoration of mitochondrial bioenergetics and ΔΨm, Ca^2+^- and cAMP-dependent secondary signalling converging on PI3K/Akt, MAPK/ERK and AMPK, redox-sensitive activation of the Nrf2/Keap1/ARE antioxidant axis, dampening of NF-κB- and NLRP3-driven inflammation, suppression of the HIF-1α/VEGF angiogenic node, anti-apoptotic remodelling of the Bcl-2/Bax balance, and PGC-1α/TFAM/NRF1-mediated mitochondrial biogenesis coupled with PINK1/Parkin-mediated mitophagy. This integrated molecular framework provides a plausible link between photophysical events at the CcO metal centres and the clinical observations reported across retinal disease. The strength of that clinical evidence is uneven: it is most secure for non-exudative AMD, where randomized controlled trials support a disease-modifying effect, whereas the data in retinitis pigmentosa, central serous chorioretinopathy and pathological myopia, together with the preclinical models of photoreceptor injury, remain preliminary and require confirmation. The recognition that mitochondrial function, and possibly PBM responsiveness, is regulated by the BMAL1/CLOCK–SIRT1–PGC-1α circadian axis adds a conceptually attractive, though still largely unproven, dimension to therapeutic design. Whether aligning the delivery of red and near-infrared light with the endogenous mitochondrial peak in the morning hours can enhance clinical efficacy is an appealing hypothesis that now needs to be tested directly in time-stratified trials. If validated, molecularly informed and chronotherapeutically optimized protocols could help to shape the next phase of PBM in ophthalmology.

## Figures and Tables

**Table 1:** The consolidated molecular targets and signalling pathways engaged by photobiomodulation in the retina. Each row summarizes one of the principal molecular axes discussed in Section 2.1, Section 2.2, Section 2.3, Section 2.4, Section 2.5, Section 2.6 and Section 2.7, listing representative targets, functional readouts and the net effect of red/near-infrared light delivery on each pathway. Arrows indicate the direction of change induced by photobiomodulation: ↑ denotes an increase, upregulation or activation, and ↓ denotes a decrease, downregulation or inhibition of the corresponding target, readout or pathway. Abbreviations: ATP, adenosine triphosphate; Δp, proton-motive force; ΔΨm, mitochondrial membrane potential; CcO, cytochrome c oxidase; ROS, reactive oxygen species; GSH, reduced glutathione.

Refs.	PBM Effect	Functional Output	Molecular Targets	Pathway/Mechanism
[3,4,15,18]	Acute increase in ATP synthesis; reversal of NO-mediated respiratory inhibition	NO photodissociation; restored electron flow at complex IV; ↑ proton-motive force (Δp); ↑ ΔΨm	Cytochrome c oxidase (CuA, CuB, heme a, heme a_3_); mitochondrial NO; structured water at proton channels	CcO photoactivation
[1,16,17,18]	Bioenergetic rescue of RPE and photoreceptors; reduced senescence markers	Sustained ATP production; recovery of ΔΨm; ↑ CcO subunit expression	Complex IV; F_1_F_0_-ATP synthase; ΔΨm	Mitochondrial bioenergetics
[12,17,20]	Renewal of mitochondrial pool; durable post-treatment bioenergetic gains	Coordinated nuclear and mitochondrial transcription; mtDNA replication; ↑ mitochondrial mass	PGC-1α; NRF1; NRF2 (nuclear respiratory factors); TFAM; mtDNA	Mitochondrial biogenesis
[1,12,17]	Quality control of the mitochondrial network; preferential elimination of dysfunctional organelles	Normalized fission/fusion balance; selective ubiquitination of damaged mitochondria; lysosomal clearance	Drp1; Mfn1/2; Opa1; PINK1; Parkin; Miro	Mitochondrial dynamics and mitophagy
[3,4,17]	Cytoprotection and neurotrophic support of inner and outer retinal neurons	Phosphorylation of CREB; induction of BDNF and BCL2 transcription	MCU; CaMKII; Ca^2+^-sensitive adenylate cyclases; PKA; EPAC; CREB	Ca^2+^/cAMP/PKA-CREB signalling
[3,4,17,19]	Anti-apoptotic and trophic signalling at therapeutic PBM doses	Phospho-inactivation of BAD; protein synthesis; pro-survival kinase signalling	PI3K; Akt; BAD; mTORC1; ERK1/2; p38 MAPK	PI3K/Akt/mTOR and MAPK/ERK
[3,12,17]	Integration of bioenergetic, redox, autophagic and circadian signals	Phosphorylation/deacetylation of PGC-1α; coupling of energy state to biogenesis and autophagy	AMPK; SIRT1; SIRT3; NAD^+^; PGC-1α; ULK1	AMPK/SIRT1/PGC-1α axis
[3,17,19]	↑ GSH; ↓ pathogenic ROS; cytoprotection against oxidative damage in RPE	Transient mitochondrial ROS burst → Keap1 oxidation → Nrf2 nuclear translocation → ARE transcription	Keap1 (Cys151/273/288); Nrf2; small Maf; ARE; HO-1; NQO1; GCLC/GCLM; SOD2; catalase; GPx; TXNRD1	Nrf2/Keap1/ARE antioxidant programme
[3,17,19,21]	Photoreceptor and RPE survival; resistance to oxidative apoptosis	↑ Bcl-2/Bax ratio; reduced cytochrome c release; ↓ caspase-3/9 activation; mPTP stabilization	Bcl-2; Bcl-xL; Mcl-1; Bax; Bak; Bid; BAD; cytochrome c; caspase-9/3; PARP; mPTP; cardiolipin	Anti-apoptotic remodelling
[3,4,17,19]	↓ TNF-α, IL-1β, IL-6, IL-18; ↓ MMP-2/9; ↓ pyroptosis in RPE and microglia	Stabilization of IκBα; ↓ NF-κB nuclear translocation; ↓ NLRP3 priming and assembly	IKK; IκBα; p65/p50 NF-κB; TLR4; NLRP3; ASC; procaspase-1; gasdermin-D	NF-κB and NLRP3 inflammasome
[4,5,6,23]	↓ drusen volume growth; ↓ incidence of geographic atrophy; resolution of subretinal fluid in CSCR	↓ HIF-1α stabilization; ↓ VEGFA transcription; restored RPE pump function	HIF-1α; HIF-1β; VEGFA promoter; Na^+^/K^+^-ATPase (RPE)	HIF-1α/VEGF axis (590 nm)
[11,12,13,14,20]	Maximal PBM efficacy in the morning (≈08:00–11:00); attenuated response in the afternoon	Time-of-day modulation of CcO activity, NAD^+^ availability and PGC-1α deacetylation	BMAL1/CLOCK; PER1–3; CRY1–2; REV-ERBα/β; NAD^+^; SIRT1/SIRT3; PGC-1α	Circadian–molecular crosstalk

## Data Availability

The original contributions presented in this study are included in the article. Further inquiries can be directed to the corresponding author.
